# Citizen science helps in the study of fungal diversity in New Jersey

**DOI:** 10.1038/s41597-022-01916-z

**Published:** 2023-01-04

**Authors:** Maria Shumskaya, Nina Filippova, Laura Lorentzen, Shazneka Blue, Carrie Andrew, Nicholas S. Lorusso

**Affiliations:** 1 Department of Biology, Kean University, 1000 Morris Ave, Union, NJ 07083 USA; 2 Yugra State University, Chekhova str., 16, Khanty-Mansiysk, 628012 Russia; 3 Oberlin College & Conservatory, Biology Department, 119 Woodland Street, Oberlin, Ohio 44074 USA; 4 Department of Natural Sciences, University of North Texas at Dallas, 7300 University Hills Blvd, Dallas, TX 75241 USA

**Keywords:** Biodiversity, Fungal ecology

## Abstract

The history of fungal diversity of the Northeastern United States is currently fragmentary and restricted to particular functional groups or limited geospatial scales. Here, we describe a unique by its size, lifespan and data originators dataset, to improve our understanding of species occurrence and distribution across the state and time. Between the years 2007 to 2019, over 30 parks and nature preserves were sampled during forays conducted by members of the New Jersey Mycological Association (USA), a nonprofit organization of fungi enthusiasts. The dataset contains over 400 000 occurrences of over 1400 species across the state, made up mostly of the phylum Basidiomycota (89%) and Ascomycota (11%), with most observations resolved at the species level (>99%). The database is georeferenced and openly accessible through the Global Biodiversity Information Facility (GBIF) repository. This dataset marks a productive endeavor to contribute to our knowledge of the biodiversity of fungi in the Northeastern United States leveraging citizen science to better resolve biodiversity of this critical and understudied kingdom.

## Background & Summary

Fungi are highly diverse and crucial for a wide variety of ecosystem services. They are one of the largest groups of decomposers, playing an essential role in nutrient cycling as saprotrophs or mycorrhiza, and facilitating ecosystem feedback to climate changes^1–4^. Fungi are a species-rich taxon of approximately 2.2–3.8 million species^5^, lesser in number to only compared with terrestrial arthropods (over 7 million species^6^). The global inventory of fungal species presents a greater challenge compared to other taxonomic groups, due to a temporal nature of bodies of the most fungi. For example, plants (ca 374 000 species^7^) have a lower estimated species number, but are much better studied and documented in a biodiversity context. While plants do play critical roles in global carbon cycling, justifying historical focus on that group, fungi play an equally important role as decomposers and nutrient cyclers. Functional groups such as the mycorrhizae are key players in regulation of carbon dioxide^8^, nitrogen and phosphorus cycling^9,10^, with up to 80% of nutrient cycling being provided via fungal symbionts. Given that much of the nutrient cycling associated with gross primary production as well as climate change (mediated through carbon dioxide consumption by plants) may be so strongly tied to fungi, understanding their diversity is critically important, making the bias against fungi a major gap in our understanding of taxa relevant to these cycles. Occasional surveys of fungal diversity based on fruiting bodies have been conducted for limited geographic scales^11^, however, more often fungi are included in larger biodiversity surveys but are disproportionally under described in resulting datasets^12,13^. While some large-scale studies of certain important fungal groups like mycorrhizae or parasitic species have been conducted^14,15^, broader evaluation of fungal biodiversity at larger spatial scales is still limited. Publicly available datasets^11,12,16–19^ of fungi provide critical data for specialists studying biodiversity, ecology, environmental science, and mycology but more data is required to access the full extent of global fungal diversity. With recent developments in DNA sequencing technologies allowing detection and description of fungal species based on DNA evidence alone, the number of fungal species is expected to rapidly grow^20^.

The limited detectability, attraction for research, and identification of fungi by both professionals and amateurs are constantly expanding. This rapidly growing inclusion of fungi into our overall study of biodiversity will only continue to expand existing DNA reference libraries^21^. Citizen science, too, takes new shapes as not only local communities, but entire mycological congresses^22^ use approaches such as BioBlitz to complement traditional surveys.

At present, nature conservation efforts for fungi as are still developing in the United States^23,24^, as prior survey initiatives have been largely scattered and maintained by voluntary efforts of resident regional mycologists (amateur or professional). Due to the fragmented nature of previous observations, there is a need to resolve fungal species diversity at regional scales in the United States, especially in highly populated areas comprised of multiple habitat and ecosystem types influenced by the legacy of citizen science initiatives^5,25,26^. For states comprised of a variety of different ecosystem types, such as New Jersey, this presents a major gap in knowledge given the degree of geographic and ecological diversity seen state-wide. While some efforts have been made to characterize fungal diversity for New Jersey, these attempts have largely been restricted to smaller geographic scales or specific taxonomic groups of interest such as lichens^27,28^ or parasitic species^29^. Despite these historical limitations, there is a pressing need to better characterize fungal biodiversity at larger spatial scales with recent work at global scales emerging for specific fungal guilds^30–34^.

The challenge presented by the lack of sufficient fungal diversity data can be resolved, to some extent, using citizen science. With the recent development of digital technologies, citizen science has been successful in contributing to fundamental research^35–37^. Several online platforms collect photographic and written observations directly from citizens (e.g. mushroomobserver.org, iNaturalist.org or fundis.org). However, the observations made via these portals are mostly recent; the tremendous efforts of amateur groups who have been tracking fungal diversity before the digital era are often left closed to the global research community due to the lack of the proper data storage and sharing protocols and so it was not uncommon for the local organizations to keep records on a personal computer or in a hand-written format. This limitation in protocols for sharing data collected by citizen scientists presents one of the major opportunities to researchers of biodiversity at larger spatial scales and making these datasets openly available is critically important to better resolve global biodiversity.

**Fig. 1:**
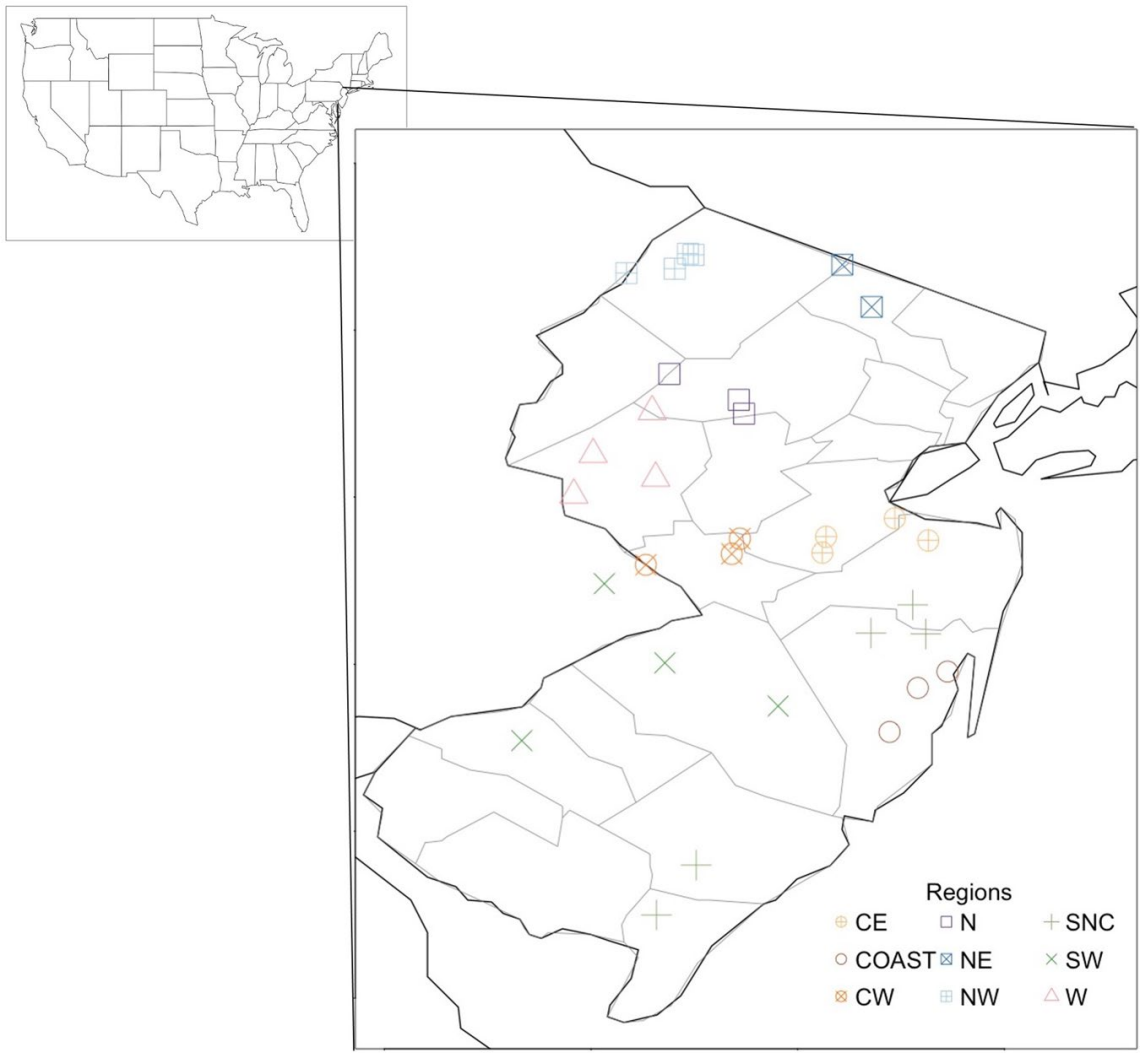
A map of the state of New Jersey, USA, where occurrence data were collected. Colored symbols: sub regions showing geographic average of the sampled sites. CE – Central East, COAST – Coastal, CW – Central West, N – North, NE – North East, NW – North West, SNC – South Non-Coastal, SW – South West, W - West.

Here, we describe a dataset consisting of fungal taxa for the state of New Jersey collected as part of citizen science forays in 32 parks and nature preserves throughout the state, separated into nine sub regions^38^ (Fig. 1), Tabel 1.

Data included in this dataset were collected between the year 2007 and 2019, by volunteers of the New Jersey Mycological Association (NJMA, www.njmyco.org) as part of their organization’s yearly sampling forays. Established in 1971 as the Lakeland Mycology Club, NJMA is now non-profit organization with over 800 members motivated by their interest in fungi, the only organization of its kind in the state of New Jersey. NJMA has amassed a wealth of citizen science data through decades of sampling events. Importantly, NJMA maintains an active herbarium of approximately 3000 vouchered specimens stored at Rutgers University in New Brunswick; however, this repository is currently not a part of the Chrysler Herbarium (CHRB) at Rutgers. Here, we showcase how large quantities of data collected across a variety of habitats and locations over a span of 12 years by volunteers has contributed to scientific knowledge in a cost-effective and data rich manner. Our interest was to prepare the collected data in a standardized format and make it open access, to increase the applicability to fungal biodiversity research. The resultant dataset is also meant to raise interest among citizen science and scientists to increase the amount of accessible data on the distribution of species^37,39^. Given that the North American Mycological Association (NAMA, https://namyco.org/clubs.php) has records of over 90 similar groups across 37 states this type of citizen science driven data collection has the potential to exponentially increase our knowledge of fungal taxa across the United States.

**Fig. 2:**
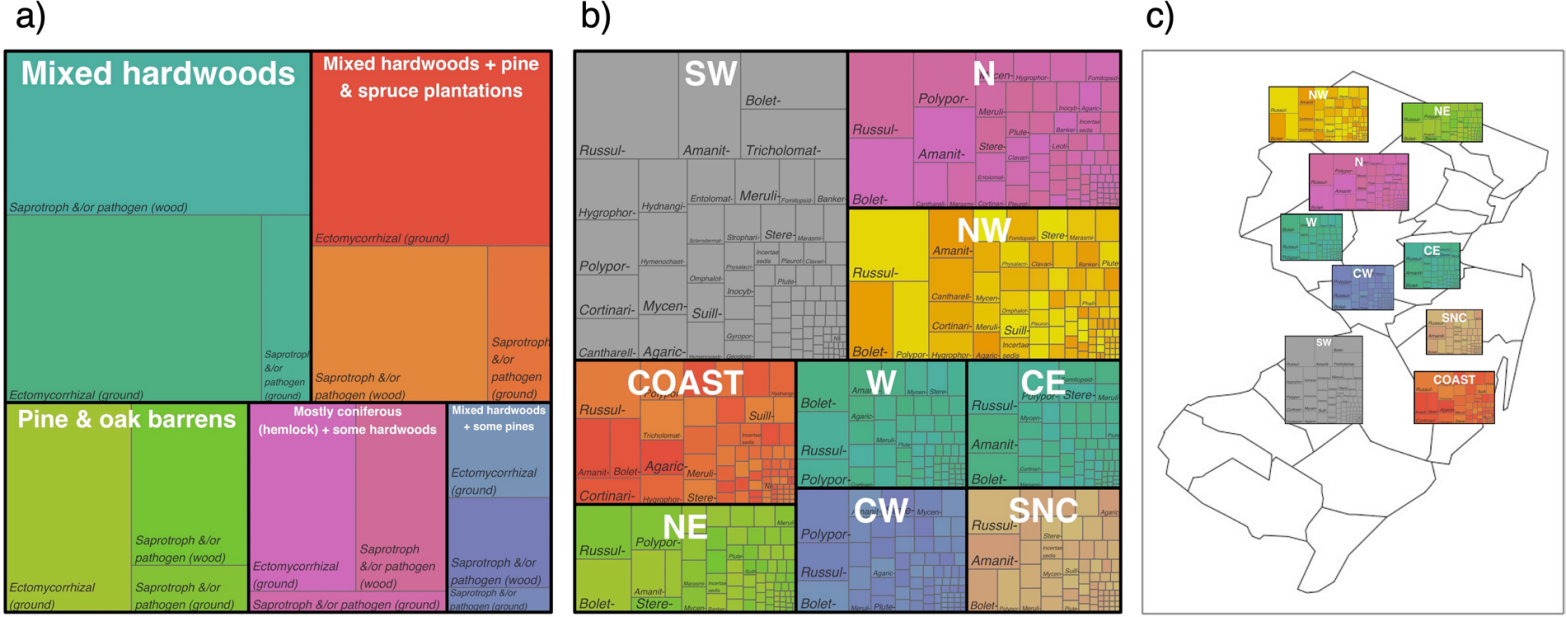
Trends for fungal taxa in the dataset. (**a**) differences in the relative proportion of common ecological guilds seen in different sample sites categorized by primary forest type. (**b**) regions sampled across the state and families of fungi found within these regions. (**c**) regions sampled across the state plotted onto a map of New Jersey and families of fungi found within these regions. In all plots the size of the boxes is proportional to the number of observations for those habitat types, regions, guilds, or functional groups. CE – Central East, COAST – Coastal, CW – Central West, N – North, NE – North East, NW – North West, SNC – South Non-Coastal, SW – South West, W - West.

The dataset presented here^38^ highlights the taxonomic diversity for the state of New Jersey from 210 surveys (corresponds to 210 records in the event table), with 400 260 records in the occurrence table. Overall, 1906 taxa with presence/absence information for each survey are published. In total, 96% of the records in the occurrence table are absence data. The taxonomic structure is presented by 2 kingdoms (Fungi and Protista for slime molds), 5 phyla, 20 classes, 58 orders, 162 families, 516 genera and 1483 species. Some species names that were assigned to the observations earlier in the data collection are now outdated, but are kept in the dataset as synonyms or under-identified taxa. Together with these records, the total number of taxa sums up to 1850. Taxa varied both by environment type based on primary forest composition (Fig. 2a) and by region (Fig. 2b), although the regional effect may also be influenced by a sampling month.

The dataset is supplemented with openly available environmental variables of interest (average region temperature; maximum region temperature; average region humidity due point; average region precipitation; average region wind; retrieved from the United States Geological Survey https://www.usgs.gov and National Weather Service https://www.weather.gov databases) for each sampling location in order to provide insight into factors contributing to changes in the distribution of taxa. This information is available for each sampling event (survey) in the dataset at GBIF.org.

**Fig. 3:**
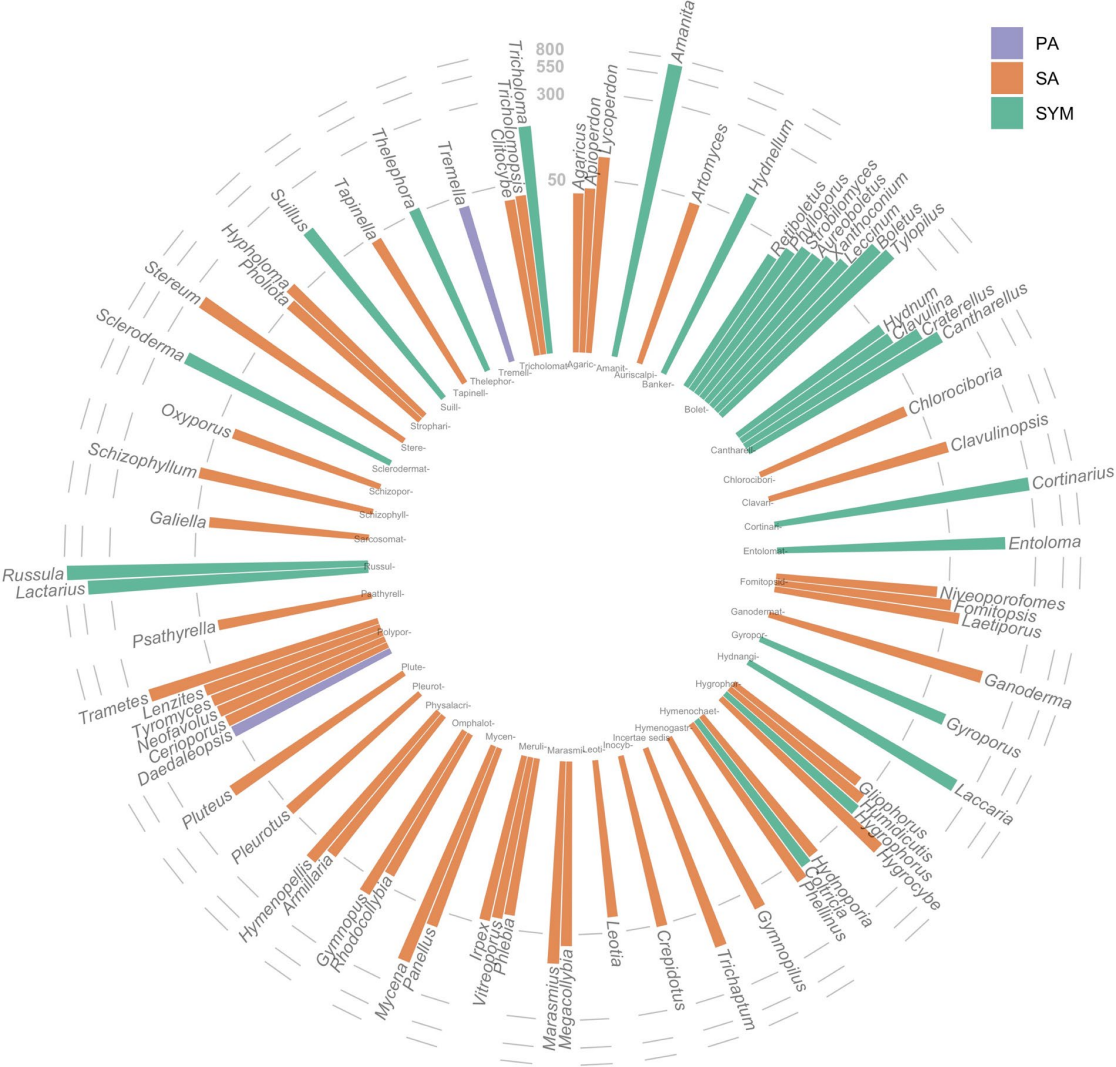
Most commonly observed taxa across all sampling sites and years shown as unscaled numbers of observations on a log_10_ scale. Colored by trophic type (sensu FunGuild;^51^ PA - pathotroph, SA - saprotroph, SYM - ectomycorrhizal symbiotroph) and sorted by the most common fungal families, with family names on the inside of the ring. Tips of each bar: names of commonly observed genera.

**Fig. 4:**
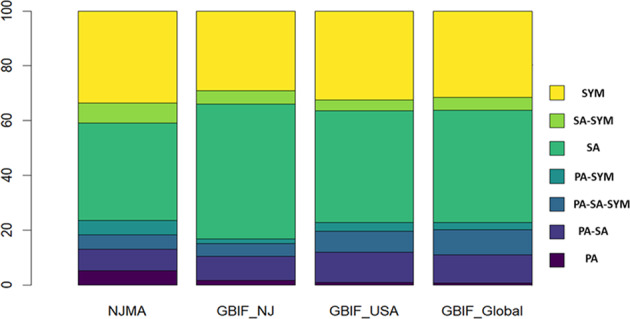
Proportion of seven guild types (sensu FunGuild^51^) in Agaricomycetes for the evaluated datasets. SYM - symbiotroph, SA- saprotroph, PA- pathotroph. NJMA: from NJMA dataset^40^, GBIF_NJ: records retrieved from GBIF.org for New Jersey^41^, GBIF_USA: records retrieved from GBIF.org for the United States^42^, GBIF_global: global records retrieved from GBIF.org^43^.

Despite the variable nature of collection across this period (such as different frequency of visiting of the same sampling sites, Table 2), this dataset presents an opportunity for researchers and citizen scientists interested in fungal biodiversity of the Northeastern United States. The dataset describes relative abundances for common taxa over time. Their trophic types are presented on Fig. 3. To expand our understanding of how this dataset compares to other similar attempts to capture fungal diversity across geographic scales, we compared our guild data to other Agaricomycetes datasets published at GBIF.org for New Jersey, the United States, and globally (Fig. 4). Despite some variability in specific species present across these spatial scales, we found similar proportions of the guild types (Fig. 4). Together, this suggests that our dataset captures information similar to other datasets of this type when considering functional roles of fungi, while adding to our knowledge of region-specific introduced or newly observed species as biodiversity changes globally (Tables 3, 4).

We suggest that this dataset, and the associated collection methods used by the New Jersey Mycological Association, could be used as a model for a systemic approach for evaluating fungal diversity across the United States. Through increasing open and digital access to fungal data, we expect that the presented dataset contributes to more complete documentation of life on Earth beyond charismatic taxa.

## Methods

### Geographic coverage

The state of New Jersey is a spatially small (22 610 km^2^) yet densely populated (8.885 million) member of the mid-Atlantic states in the Northeastern United States. Comprised of a number of ecosystem types, including coastal regions to the east, deciduous forests in the central and northern parts of the state, and pine forests/barrens in the south, New Jersey presents a diversity of ecological community types. The state ranges between sea level and 550 meters and is flat with an average elevation of only 69 meters. The state is also marked by a high population density and degree of human use, with roughly a third of total land for the state currently developed. The state saw continuous development during the sampling duration for the dataset with an increase of more than 360 000 acres of land being developed between the years 1986 and 2015 (NJDEP, 2020, https://www.nj.gov/dep/dsr/trends/). The climate follows four regular seasons typical of the Northeastern United States with most of the state characterized as humid subtropical. Average rainfall ranges between 1016–1295 mm. Average yearly temperature is roughly 13.3 °C. Both average temperature and precipitation have been recently increasing, consistent with climate change.

### Study extent

Between 2007 and 2019, the NJMA conducted yearly surveys, where members would visit parks across the state at approximately the same time each year to conduct sampling forays. Most sampling sites were parks or nature preserves (Table 1) selected due to their probability of having representative fungal biodiversity for that part of the state. Samples for the foray locations were collected for identification from within the boundaries of the sampling sites.

**Table 1:** Localities sampled as part of NJMA yearly forays with representative coordinates, generalized habitat, and user defined regions within the state.

Sampling Locality	Latitude	Longitude	Generalized Habitat	Region
Cheesequake State Park	40.43615	−74.2653	Mixed hardwoods	CE
Helmetta bog	40.38147	−74.4312	Mixed hardwoods	CE
Holmdel County Park	40.37041	−74.1843	Mixed hardwoods	CE
Thompson Park	40.33403	−74.4403	Mixed hardwoods	CE
Cattus Island Park	39.97802	−74.138	Pine & oak barrens	COAST
Jakes Branch County Park	39.92915	−74.2096	Pine & oak barrens	COAST
Wells Mills County Park	39.79729	−74.278	Pine & oak barrens	COAST
Herrontown Woods	40.37568	−74.6397	Mixed hardwoods	CW
Princeton Institute Woods	40.33075	−74.6594	Mixed hardwoods	CW
Washington Crossing State Park	40.29818	−74.8672	Mixed hardwoods + pine & spruce plantations	CW
Meadowood Park	40.79138	−74.6424	Mixed hardwoods	N
Schiff Nature Preserve	40.75003	−74.6298	Mixed hardwoods	N
Stephens State Park	40.86891	−74.8102	Mixed hardwoods	N
New Weis Center for Education, Arts, and Recreation	41.06979	−74.3211	Mixed woods	NE
Wawayanda State Park	41.19632	−74.392	Mixed woods (hemlock stands, mixed hardwoods)	NE
Pocono Environmental Education Center	41.17124	−74.9144	Mostly coniferous (hemlock) + some hardwoods	NW
Stokes State Forest − Kittle Field	41.18439	−74.7973	Mostly coniferous (hemlock) + some hardwoods	NW
Stokes State Forest − NJ School of Conservation	41.22571	−74.7518	Mostly coniferous (hemlock) + some hardwoods	NW
Stokes State Forest, Lake Ocquittunk	41.2294	−74.7652	Mostly coniferous (hemlock) + some hardwoods	NW
Belleplain State Forest	39.24875	−74.8415	Pine barrens	SNC
Estell Manor Park	39.3983	−74.7454	Pine barrens	SNC
Forest Resource Education Center	40.09304	−74.3231	Pine & oak barrens	SNC
Manasquan Reservoir Environmental Center	40.17787	−74.2221	Mixed woods/oak barrens	SNC
Ocean County Park	40.09062	−74.1903	Pine & oak barrens	SNC
Brendan T. Byrne Park and State Forest	39.87479	−74.5474	Pine barrens	SW
Chestnut Branch Park	39.77129	−75.1672	Mixed hardwoods + some pines (?)	SW
Franklin Parker Preserve	40.24178	−74.9678	Pine & oak barrens	SW
Rancocas State Park	40.0041	−74.821	Pine & oak barrens	SW
Deer Path Park	40.55654	−74.8434	Mixed hardwoods	W
Hoffman County Park	40.62797	−74.9949	Mixed hardwoods (mostly oak)	W
Horseshoe Bend Park	40.50603	−75.0415	Mixed hardwoods	W
Teetertown Ravine Nature Preserve and Crystal Springs	40.75654	−74.8518	Mixed hardwoods	W

CE – Central East, COAST – Coastal, CW – Central West, N – North, NE – North East, NW – North West, SNC – South Non-Coastal, SW – South West, W - West.

Sampling sites were distributed across the entire state of New Jersey (Fig. 1) and were visited with some temporal variability within each year. Some foray locations within the dataset were unique to only part of the 12-year sampling window, while other foray locations were sampled consistently across the entire sampling period (e.g. Pocono Environmental Education Center vs Wawayanda State Park, Table 2). Sites were sampled between May and November of each year with citizen scientists sampling one site per day. Sites were normally sampled during the same month across years, though some variation in sampling time did occur. To better describe similar regions across the state, we assigned regional identifications to each foray sampling locality based on habitat similarity and available climatological data. The sites sampled were selected to provide a measure of the fungal biodiversity within different ecosystems types representative of the state. Forays stopped in 2020 due to the COVID-19 pandemic and data after 2019 were not included due to changes in sampling activity.

**Table 2:** Sampling localities organized by both the region and the year of sampling (2007–2019).

Sampling locality by region	2007	2008	2009	2010	2011	2012	2013	2014	2015	2016	2017	2018	2019
Central East	•	•	•	•	•		•	•	•				
Cheesequake State Park												•	
Helmetta bog						•	•	•	•				
Holmdel County Park									•	•	•	•	•
Thompson Park													
Coastal													
Cattus Island Park		•	•	•	•	•				•		•	•
Jakes Branch County Park	•	•	•	•		•	•	•	•	•	•	•	•
Wells Mills County Park	•	•		•	•	•	•	•					
Central West													
Herrontown Woods	•	•	•										
Princeton Institute Woods	•	•	•	•		•	•	•	•	•	•	•	•
Washington Crossing State Park	•	•		•	•	•	•	•					
North													
Meadowood Park	•	•	•	•	•	•	•	•	•	•	•		•
Schiff Nature Preserve	•	•	•	•	•	•	•	•				•	
Stephens State Park	•	•	•	•		•	•		•	•	•	•	•
North East													
New Weis Center for Education, Arts, and Recreation											•	•	
Wawayanda State Park			•	•	•	•	•	•	•	•	•	•	•
North West													
Pocono Environmental Education Center	•							•					
Stokes State Forest-Kittle Field -	•	•	•	•	•	•	•	•	•	•	•	•	•
Stokes State Forest–NJ School of Conservation												•	•
Stokes State Forest, Lake Ocquittunk			•	•	•		•	•	•	•	•		•
South Non-Coastal													
Belleplain State Forest									•	•	•	•	•
Estell Manor Park												•	•
Forest Resource Education Center										•	•	•	•
Manasquan Reservoir Environmental Center	•	•	•	•	•	•	•	•	•				
Ocean County Park											•		
South West													
Brendan T. Byrne Park and State Forest	•	•	•	•	•		•	•	•	•	•	•	
Chestnut Branch Park													•
Franklin Parker Preserve			•	•	•	•		•	•	•	•		
Rancocas State Park	•	•	•	•	•	•				•	•		
West													
Deer Path Park			•										
Hoffman County Park	•	•	•	•	•	•	•	•	•				
Horseshoe Bend Park								•				•	
Teetertown Ravine Nature Preserve and Crystal Springs											•	•	•

### Data acquisition

The established sampling foray method has been practiced by NJMA for the past 30 years. Sampling forays were conducted for two hours at each foray location with any member able to participate in collection. Some forays were made open to the public and participant numbers ranged from 5 to 30 people, with all participants starting from specific starting point. Within the two-hour foray period, samplers surveyed approximately a one-mile radius around the starting point and collected any visible sporocarps and returned them to foray leaders for identification. Hypogeous taxa were not explicitly sampled as part of these forays, and the focus was on macroscopic fruit bodies (with select observations of micro fungi). Sampled taxa were identified on site by foray leaders, and records were stored in the NJMA documentation archive with some samples stored in the herbarium. This combined strategy, using experts for identification and many participants for sample collection, effectively leveraged citizen science to make use of many samplers without formal scientific training in collection when a limited number of visiting taxonomic experts are available. The lists of observed species were recorded and saved as hand-written, PDF or Microsoft Office documents and stored at personal computers of NJMA members. A summary result of the forays was published in a PDF e-letter from the Association to its members and also shared on their website www.njmyco.org.

Datasets for different regional scales used in Fig. 4 and Table 4 were retrieved from GBIF.org and checked for accuracy to ensure species names matched across regional lists. A list of Agaricomycetes, a class highly represented in the NJMA dataset^40^, was selected from the dataset and used to compare diversity at higher spatial scales. Checklists for preserved specimens within the Agaricomycetes were retrieved (filtered by basis of record – “preservedSpecimen”, occurrence status = “present”) for three regional scales: New Jersey^41^, the United States^42^, and global^43^.

**Table 3:** Species counts for Agaricomycetes datasets published at GBIF.org.

Dataset name (shared/unique species)	NJMA	GBIF NJ	GBIF USA	GBIF Global
**NJMA**	**1248 species**	NJMA: 691 unique GBIF NJ: 605 unique Shared: 557	NJMA: 32 unique GBIF USA: 7636 unique Shared: 1216	NJMA: 4 unique GBIF Global: 28349 unique Shared: 1244
**GBIF NJ**		**1162 species**	GBIF NJ: 0 unique GBIF USA: 7690 unique Shared: 1162	GBIF NJ: 0 unique GBIF Global: 28431 unique Shared: 1162
**GBIF USA**			**8852 species**	GBIF USA: 0 unique GBIF USA: 20741 unique Shared: 8852
**GBIF Global**				**29592 species**

NJMA: from the dataset of this study^40^. GBIF NJ: the dataset of preserved specimens for New Jersey region^41^. GBIF USA: the dataset of preserved specimens for the USA^42^. GBIF Global: global records of preserved specimens^43^. Total species counts are presented on the diagonal. Unique or shared species numbers are presented above the diagonal.

**Table 4:** Number of occurrences per dataset published in GBIF.org.

Dataset	Number of observations	Percentage of observations from total number of global observations
Artportalen (Swedish Species Observation System)	1661121	24
Observation.org, Nature data from around the World	747761	11
Danish Mycological Society, fungal records database	518310	7
Norwegian Species Observation Service	494795	7
iNaturalist Research-grade Observations	426203	6
Fungi of parks, forests and reserves of New Jersey (2007–2019)	400260	6
Swiss National Fungi Databank	271159	4
Österreichische Mykologische Gesellschaft - Austrian Mycological Society	163957	2
BLS Lichen Database: England 1650–2016	162753	2
BLS Lichen Database: Scotland 1700–2016	119995	2
Others	2090943	30

Data were searched globally (using filters: kingdom - “fungi”, basis of record - “human observation”, occurrence status - “everything”, and year - “2007–2019”. Total number of observations found: 6 194 448.

### Taxonomic identification

Initial identification of taxa collected during forays was completed by foray leaders in the field using existing literature (listed in the GBIF repository^43^) by assigning species names of the closest morphospecies.

### Data digitalization and unification

The species lists were obtained from various NJMA members and converted from the existing format (Word, PDF, Excel) to Excel-based templates compatible with the EarthCape database (https://earthcape.com/,^44^), spell checking, formatting, association of data with information fields such as locality name or scientific taxon name was carefully performed. EarthCape allowed consolidation of locations into different user defined regions according to geographic location, habitat type, or climatic zone. The EarthCape database also confirmed consistent taxonomic synonymizing by comparison of user-assigned species identities against currently accepted taxonomic names of GBIF taxonomic backbone at GBIF.org, and allowed to convert the data to the GBIF format to prepare for the dataset publication.

## Data Records

The dataset contains a description of whether a species (or in rare cases, a genus) were observed during a particular foray event. For all taxa observed across all forays, the presence or absence of that taxa is recorded in a particular foray and supplemented by the foray time and location, geographic data (coordinates, region, etc.), habitat type based on dominant hardwood in that location, and climatic variables (averages for temperature, precipitation, wind speed, dew point). Data on soil chemistry and geological variables were retrieved from United States Geological Survey (https://www.usgs.gov). Data for climate variables were retrieved from National Weather Service (www.weather.com) with representative collection stations identified and used for each region.

Our database is stored locally and is freely accessible through the GBIF (Global Biodiversity Information Facility) repository (www.gbif.org) under the 10.15468/7scek4^38^. For each occurrence record there are 61 fields of information, recorded using terms of Darwin Core standard (DwC)^45^ (http://rs.tdwg.org/dwc/terms). The database includes a supplemental data table that provides the climatological and geological data for each foray. These “Measurement or fact” extension table can be downloaded together with the source data. The dataset will be updated as new yearly forays occur to keep data consistent across forays. It is our intent that this data collection, made possible by the common interests among citizen science and scientists, continues to expand our knowledge of fungal distribution and biodiversity.

## Technical Validation

The data was validated using standardized procedure for digitization, formatting, and content checking of the occurrence as in earlier studies^46^. Integration and digitizing of data from various resources (electronic files, hand written documents etc.) was performed using EarthCape database software^44^ built-in validation tools such as formatting and spelling checks, linked tables, alignment of nomenclature with the GBIF backbone^47^, and synonymizing. To ensure the names and authors for all taxa observed, species names were confirmed using Index Fungorum (http://www.indexfungorum.org). Homotypic names were checked to refer to the accepted names. Next, the data was exported into three linked tables using Darwin Core standard^45^: occurrence, event and measurementOrFact. The final data cleaning and processing was made using Linux command line scripts using bash and awk by R. Mesibov^48^, and included structure, format and content of data. All data were checked again once prepared for publication via GBIF to validate the taxonomy, climate data, and the occurrence status. Our dataset was compared to similar records, as well as records at larger spatial scales, from GBIF.org (Table 3). We confirmed that all species from our dataset were captured at larger scales, with only several unique observations. When compared to the GBIF data available for New Jersey, our dataset shows 557 shared species. The difference with 691 species unique to GBIF records for New Jersey and 605 species unique to our dataset likely results from the study focus, regions sampled, and changes in the fungal composition of the state over the years. Our dataset also competes with some of the largest datasets for fungal biodiversity in GBIF, contributing significantly to the global data pool (6% of global fungal occurrences for the period of 2007–2019) (Table 4). Consistency of the taxonomic names was managed using GBIF Species API (https://www.gbif.org/developer/species) and the rgbif R package^49,50^. Trophic type was assigned using R package funguild^51^.

## Usage Notes

We suggest that the data within this dataset be used by researchers interested in evaluating large scale changes in biodiversity for fungi across space and time as well as researchers interested in studying the ranges of particular fungal taxa or guilds, for example, in remote sensing of mycorrhizal composition^52^. Beyond usage by formal researchers, we suggest our methods to be used by citizen science groups collaborating with universities and data repositories to make this data more accessible. The described method have proven to be efficient at leveraging citizen science records for fungal biodiversity and so we implore similar groups to consider reviewing our dataset with the included collection methods and planning their own forays using similar strategies. By leveraging shared collection methods across enthusiast societies with platforms for sharing data like GBIF and iNaturalist, we can greatly improve knowledge of fungal biodiversity across larger spatial scales.

## Data Availability

Figures were prepared using R Statistical Software (v4.1.2; R Core Team 2021)^53^, packages ggplot2^54^, treemap^55^,funguild^51^ and fungarium^56^. The dataset was shared via GBIF.org using Integrated Publishing Toolkit (IPT, www.gbif.org/ipt)^57^. No original code was created to generate the dataset.
